# Practical Assessment of an Interdisciplinary Bacteriophage Delivery Pipeline for Personalized Therapy of Gram-Negative Bacterial Infections

**DOI:** 10.3390/ph15020186

**Published:** 2022-02-02

**Authors:** Silvia Würstle, Jana Stender, Jens André Hammerl, Kilian Vogele, Kathrin Rothe, Christian Willy, Joachim Jakob Bugert

**Affiliations:** 1 Department of Internal Medicine II, School of Medicine, University Hospital Rechts der Isar, Technical University of Munich, 81675 Munich, Germany; 2 Bundeswehr Institute of Microbiology, 80937 Munich, Germany; JanaStender@bundeswehr.org; 3 Unit Epidemiology, Zoonoses and Antimicrobial Resistances, Department Biological Safety, German Federal Institute for Risk Assessment, 10589 Berlin, Germany; Jens-Andre.Hammerl@bfr.bund.de; 4 Physics of Synthetic Biological Systems-E14, Physics-Department and ZNN, Technical University Munich, 85748 Munich, Germany; kilian.vogele@gmail.com; 5 Institute for Medical Microbiology, Immunology and Hygiene, School of Medicine, Technical University of Munich, 81675 Munich, Germany; kathrin.rothe@tum.de; 6 Trauma & Orthopaedic Surgery, Septic & Reconstructive Surgery, Research and Treatment Centre Septic Defect Wounds, Bundeswehr (Military) Academic Hospital Berlin, 10115 Berlin, Germany; christianwilly@bundeswehr.org

**Keywords:** bacteriophage, phage therapy, antimicrobial resistance, COVID-19 superinfection, *Klebsiella* spp., cell-free extract, in vitro phage production

## Abstract

Despite numerous advances in personalized phage therapy, smooth logistics are challenging, particularly for multidrug-resistant Gram-negative bacterial infections requiring high numbers of specific lytic phages. We conducted this study to pave the way for efficient logistics for critically ill patients by (1) closely examining and improving a current pipeline under realistic conditions, (2) offering guidelines for each step, leading to safe and high-quality phage supplies, and (3) providing a tool to evaluate the pipeline’s efficiency. Due to varying stipulations for quality and safety in different countries, we focused the pipeline on all steps up to a required phage product by a cell-free extract system. The first of three study runs included patients with respiratory bacterial infections from four intensive care units, and it revealed a cumulative time of up to 23 days. Ultimately, adjustment of specific set points of the vulnerable components of the pipeline, phage isolation, and titration increased the pipeline’s efficiency by 15% and decreased the maximum required time to 13 days. We present a site-independent practical approach to establish and optimize pipelines for personalized phage delivery, the co-organization of pipeline components between different institutions, non-binding guidelines for every step, and an efficiency check for phage laboratories.

## 1. Introduction

In the wake of increasing antibiotic resistance, bacteriophages (phages) have gained attention as an adjuvant therapy for infections with multi-antibiotic-resistant bacteria in community-acquired and nosocomial bacterial pneumonia. Recently, phages have been used for the treatment of bacterial superinfections in COVID-19 [1,2,3]. Under the umbrella of compassionate use, more than 2000 patients have been treated with phages, including those targeting *Klebsiella pneumoniae* [4,5,6,7,8,9,10]. Several highly relevant clinical trials are underway, and approved phage composites with market authorization may provide access to phage therapy for important applications in the future. However, these static composites can only successfully target a few individual infections, due to the large number of highly specific phages and their potential genetic volatility [1,6,11,12,13,14,15]. For personalized phage therapy, international phage banks, in line with high quality standards, such as good manufacturing practice (GMP), should be substantially expanded, despite high costs, as phage banks currently have far from sufficient numbers of therapeutically needed phages for patients [12,16]. Due to the large number of wild type phages required, genetic volatility that sometimes occurs, and large cost of GMP certification, there is currently, and often will continue to be, no therapeutic offering with GMP-phages.

We conducted this study to present a phage delivery pipeline for *de novo* phage isolation for personalized and high-quality phage therapy. The described pipeline cuts the time required under realistic conditions almost in half. This will be especially useful for critically ill patients suffering from multidrug-resistant Gram-negative bacteria (MRGN). We incorporated the published isolation and purification protocols and, for the first time, describe, in detail, the interdisciplinary and logistic workflows of each step, including nine non-binding guidelines for all involved competency areas [11,12,17,18]. This study bridges gaps in the practical approach between bacterial isolation and characterization, phage isolation, phage characterization, phage production, and the interaction of various institutions, leading to timely outcomes and ways to improve them.

## 2. Results

### 2.1. Pipeline Set-Up

**Figure 1:**
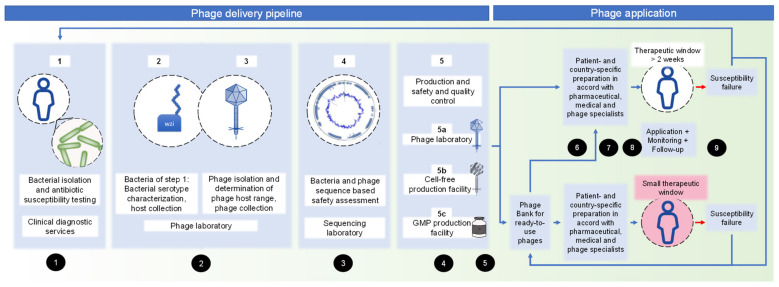
Pipeline of personalized phage delivery from an organizational perspective, considering different competencies and production sites. Numbers in black circles refer to the non-binding guidelines, Section 3.5 of this manuscript.

After clinical request for phage therapy, the different competencies required for embedding phage isolation for individual patients were assessed (Figure 1 and 3.5, non-binding guidelines). It should be noted that different competencies can be combined at one centre, or even in one person. Starting from clinical diagnostic services (step 1), the delivery pipeline covers a phage laboratory (step 2 and 3), sequencing facility (step 4), and production site (step 5). The production site offers different choices: naturally isolated phages (step 5a) for personalized therapy might be produced in a phage laboratory, as previously described [16,19]. Another production choice (step 5b) is the cell-free phage production, which describes the in vitro syntheses of phages by a cell-free transcription-translation toolbox, derived from *E. coli* [20,21]. This method offers the advantage of high-titre and clean phages; however, it is not feasible for all phages. GMP phages (step 5c) must be planned long in advance, together with a GMP facility specialized in phages. Step 5a–c can be combined according to country- and patient-specific requirements.

### 2.2. Pipeline Performance

To examine the pipeline steps important for phage laboratories, we analysed respiratory samples from four different intensive care units (ICU) of a tertiary care hospital in a high-throughput setting during phase I of this study.

Step 1: The first step revealed a total of 44 bacterial isolates from 26 different patients with tracheal or bronchial (super-)infections in the ICU during phase I. However, one patient (two different isolates) was excluded from further steps, due to missing information about the isolation location, leading to success for patient data retrieval of 95.5% of all isolates (42/44). The cohort of the ICU patients is characterized as described in Table S1. Most bacteria were isolated from COVID-19 patients (34/42 bacteria, 17/25 patients). The mortality rate of all COVID-19 ICU patients suffering from different bacterial superinfections was 52.9% (9/17). Enterobacterales were predominant in all patients (40/42). *Klebsiella* spp. (40.5%, 17/42), particularly *K. oxytoca* (7/42, 16.7%) and *K. pneumoniae* (6/42, 14.3%), were the most frequently isolated superinfecting bacteria. The next most isolated Gram-negative bacteria were *Escherichia* spp. (8/42). The bacterial resistance patterns to antibiotics are detailed in Table S2. Culturing and identification of the bacterial isolate in step 1 required an average of 24 h, and the assessment of the resistance pattern required another 24 h. Infections with polymicrobial spectrum took up to an additional 24 h, until the final report was finished.

Step 2: Bacterial characteristics of the bacteria, isolated in step 1, were entered into a database, and *Klebsiella* spp. were further characterized via capsule typing (step 2–48 h). The *wzi* capsule structures of *K. pneumoniae* are depicted in Figure S1. All MRGN were added to an existing MRGN and phage collection. Based on chronological order, with our focus on *Klebsiella* spp., 23 bacteria were transferred to phage isolation (step 3).

Step 3: Phage isolation from wastewater of a sewage treatment plant revealed a 100% discovery rate of matching phages for all bacteria. Of these, 15.4% (10/65 phages) could not be amplified to a high titre, resulting in a matching rate of 87.0% (20/23 bacteria, detailed in Table S3). Based on hands-on time and necessary waiting periods, phage isolation from sewage resulted in a cumulative time of 48–336 h. On average, 2.4 lytic, matching, and high-titre phages could be offered for each bacterium in step 3.

Step 4: All high-titre phage lysates (*n* = 55) were subjected to phage DNA extraction and Illumina short-read sequencing for an in-depth characterization and safety evaluation. For the majority of sequenced phage genomes (87.3%, 48/55), highly reliable genome sequences were derived from sequencing data, leading to a sequencing depth of 250 to >1000 per phage genome. Of the 55 samples, one sample yielded no phage-associated sequences, and one sample yielded only a partial phage sequence (*Ackermannviridae*). For five samples only lysates of 10^7^ plaque forming units (PFU)/mL were provided, and they did not yield enough DNA material for sequencing. The reason for not obtaining phage sequences might be phages based on RNA or ssDNA, modified DNA bases, or underrepresentation due to bacterial contamination. The remaining 48 sequenced phages were assigned to 30 reference phages of the National Center for Biotechnology Information (NCBI) database (last request date: 8 October 2021, see Table S4), representing the viral families *Autographiviridae* (*n* = 28), *Myoviridae* (*n* = 11), *Drexlerviridae* (*n* = 6), *Siphoviridae* (*n* = 1), *Demerecviridae* (*n* = 1), and one unclassified family. The sequenced phage genomes predominantly matched the reference phages, i.e., *Klebsiella* phage NTUH-K2044-K1-1 (*n* = 7, *Autographiviridae*) and Escherichia phage LM33_P1 (*n* = 5, *Autographiviridae*). Despite a close relationship (i.e., genome coverage 49–99% and sequence identity >90%) to their reference phages, the sequenced phages exhibited specific sequence alterations, or they significantly differed in their genomic composition. A total of six different groups, represented by genomic sizes of (i) 38–41 kb (*n* = 18), (ii) 43–45 kb (*n* = 9), (iii) 48–51 kb (*n* = 6), (iv) 109–143 kb (*n* = 4) (v), and (vi) 167–176 kb (*n* = 8), were detected. *De novo* assembly of three sequencing datasets resulted in incomplete phage genomes that were represented by the occurrence of two sequence contigs, thus preventing a reliable evaluation of the final genome size. Importantly, none of the determined phage genomes exhibited coding sequences, potentially leading to predictable, undesired effects during use, such us transferring new virulence factors (last request date: 8 October 2021). However, since the evaluation is only based on the current knowledge of specific factors, such as antimicrobial/biocide/metal resistance determinants, virulence factors, genes with allergenic potential, and toxins, the safety assessment needs to be re-evaluated from time to time, as well as at a final time before using the phages for therapeutic applications. Regarding the time required for step 4, a relatively standardized time of 72 h is needed from phage DNA isolation to safety assessment.

**Figure 2:**
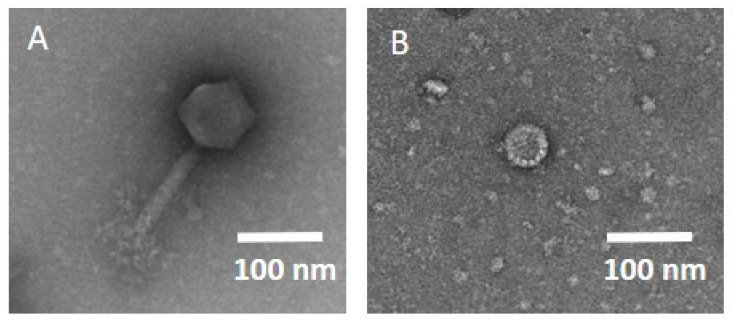
TEM image of the phages produced in the cell-free extract system. (**A**) Phage vB_EcM_muc122-IMB, host *E. coli*, myovirus, genome size 166.6 kb. (**B**) Phage vB_KoP_muc139-IMB, host *K. oxytoca*, family *Autographiviridae*, genome size 43.4 kb.

Step 5: We decided on phage production using a cell-free extract system (step 5b) to obtain phages of high-quality and -titre, without the required endotoxin removal steps, and we opted for natural phages (step 5a), where cell-free production was not possible. Successful cell-free phage assembly was tested with plaque assays, confirming that two phages, one myovirus (host *E. coli*, 166kb) and one *Autographiviridae* (host *K. oxytoca*, 43kb), could be successfully expressed, even without modifications to the cell-free extract system. Additionally, one phage against *K. pneumoniae* was successfully expressed in the cell-free system; however, the isolation location of the bacterium was not in the respiratory tract; therefore, the phage was not studied further. The final titre of all phages of step 5 was 1 *×* 10^10^ PFU, with a volume of 11 mL, and the required time for production was 10 h. The transmission electron microscopy (TEM) images can be viewed in Figure 2.

### 2.3. Pipeline Fefinement

Steps 4 and 5 met the demands for the phage delivery well. The final volume of the produced phages in the cell-free extract system could easily be increased (additional 10 h per 10 mL, simultaneous production of different phages). The preceding steps, in particular, the time requirements for step 3 (48–336 h, see Table 1), could be improved. Hence, we conducted a second study phase, with the following adaptations:

- Only *Klebsiella* spp. were included in phase II to increase the ratio of manpower to isolates.
- Apart from phage isolation from sewage, the laboratory’s phage collection was screened for matching phages, specific to bacteria targeted in this study.
- The number of tested phages from the laboratory’s phage collection was limited to the ones known to be specific for the assessed capsule type.
- The number of titration cycles was limited to two titrations.

During phase II, a total of 34 bacteria were isolated from 30 different patients, and they were forwarded to the phage laboratory. All *Klebsiella* spp. were characterized as *K. pneumoniae*. All patient data was retrieved. The collection sites, as well as *wzi* capsule types, are detailed in Table S1 and Figure S1. Phage isolation was successful for 79.4% (27/34) of the bacteria in phase II, and phages (*n* = 60) could be amplified to a high-titre for further use for 58.8% of the bacteria (20/34). In summary, the above-mentioned adaptions successfully reduced the time required for step 3 to 24–72 h. However, we observed a decrease of bacterial isolates with high-titre matching phages from 87.0% to 58.8%, and a decrease in the number of phages per bacteria (from 2.4 to 1.1 phages per bacterium for further use). The main cause of the decreases could be attributed to the limited number of plaque purification cycles, which was subsequently raised to three cycles again.

To test the refined pipeline with the adaptions mentioned above with three titration cycles, as well as in the COVID-19 peak occupancy rate in a clinic and lockdown situation, a short third collection period was initiated in the ICU. During five days, six *Klebsiella* spp. isolates from six different patients could be transferred to the phage laboratory, and 100% of patient data could be retrieved. The collection sites, as well as *wzi* capsule types, are described in detail in Table S1 and Figure S1. Titration succeeded for five of the six bacterial isolates (83.3%). Our pipeline refinement resulted in an improved number of different high-titre phages (*n* = 12), cultivated per isolate (2.0), as well as an improved cumulative time (202–298 h).

### 2.4. Efficiency Equation

Phage laboratories can compare their efficiency in providing phages for country-specific quality control for future phage therapies. This is particularly useful when attempting to optimize the pipeline or comparing the efficiency of an existing pipeline for different bacterial species. For this purpose, we have developed an efficiency equation (Equation (1) and corresponding web application to facilitate the calculation [22]. The efficiency is referred to as “E-factor”. The inputs in Equation (1) are described in Table 1 for our three study runs. Additionally, we made three assumptions regarding desired time frame, volume, and titre, which are described in the Supplementary Materials. The E factor for phase III was 0.64–0.86; compared to phases I and II, its mean value increased by 15% and 159%, respectively. The maximum time from step 1 to step 5 was reduced by 45%, due to pipeline refinement.

(1) $$\begin{array}{c} E-\mathrm{factor}=\mathrm{Percentage}\;\mathrm{of}\;\mathrm{successfully}\;\mathrm{retrieved}\;\mathrm{patient}\;\mathrm{data}\;\times \\ \mathrm{Percentage}\;\mathrm{of}\;\mathrm{bacteria}\;\mathrm{with}\;\mathrm{high}\;\mathrm{titre}\;\mathrm{phages}\;\times \\ \mathrm{Number}\;\mathrm{of}\;\mathrm{high}\;\mathrm{titre}\;\mathrm{phages}\;\mathrm{per}\;\mathrm{bacteria}\;\times \\ \mathrm{Percentage}\;\mathrm{of}\;\mathrm{reliable}\;\mathrm{genome}\;\mathrm{sequences}\;\times \frac{\mathrm{desired}\;\mathrm{time}\;\mathrm{period}}{\mathrm{actual}\;\mathrm{cumulative}\;\mathrm{time}}\times \\ \frac{\mathrm{actual}\;\mathrm{final}\;\mathrm{phage}\;\mathrm{titre}}{\mathrm{required}\;\mathrm{final}\;\mathrm{phage}\;\mathrm{titre}}\times \frac{\mathrm{actual}\;\mathrm{final}\;\mathrm{volume}}{\mathrm{required}\;\mathrm{final}\;\mathrm{volume}} \end{array}$$

Equation (1): Efficiency calculation of a phage delivery pipeline.

## 3. Discussion

### 3.1. Need for the Study

In this study, we aimed to assess phage delivery for personalized therapy in a well-structured interdisciplinary pipeline to assess and increase the pipeline’s efficiency and provide guidelines for each single step for non-phage experts. To date, ‘sur-mesure’ phage preparations have mostly been used in phage therapy, due to the limited availability of ‘prêt-à-porter’ phage products, high number of required phages, and need for rapid adaptation to emerging phage resistance during treatment [5,6,7,13,14,23]. The Belgian monograph on the use of magistral phages is a frequently encouraged step towards the implementation of a new regulatory framework for ‘sur-mesure’ phages [1,14,24,25]. Phage banks for personalized use of phages incorporate high-quality phage preparations, or phages meeting GMP-standards, but these banks have far from sufficient numbers of therapeutically needed phages for patients [12,16]. For *de novo* isolated ‘sur-mesure’ phages, the therapeutic window (the period of time during which a particular treatment can be reasonably applied to a patient) must be open for at least several weeks [5,7,13]. However, critically ill patients, such as ICU patients suffering from respiratory MRGN superinfections, require safe and rapid therapeutic offers. This is only feasible with an already established and validated pipeline guiding physicians, who are non-phage experts, through each single step (Section 3.5), as presented in Figure 1.

### 3.2. Pipeline and Guideline Set-Up

The assembly and optimization of a frictionless pipeline, in this study, focused on the most critical steps, following a clinical request for phage therapy. While it is important to examine the efficiency of the quality control process, this is beyond the scope of this study, since the procedures are country-specific. Nevertheless, guideline No. 5 (Section 3.5) summarizes the important steps of safety and quality control from a non-regulative but phage biology and clinical perspective, which has also been described in detail previously [11,19]. For the production step 5, we chose the relatively new method of cell-free extract system phage production for this study, which guarantees high-titre and quality, in terms of endotoxin or residues in the phage lysate.

### 3.3. Pipeline Refinement

Phase I represented a time-consuming process of phage isolation, whereas phase II not only shortened the overall time but also reduced the phages per bacteria for further use, due to reduced phage enrichment rounds. The E factor was the highest in phase III, representing a balance between the time spent and phages successfully matched and amplified. A higher number of phages for further use per bacterium could be suitable if the phages or phage cocktails are to be rotated during a long treatment period to counter resistance development.

We conclude that, due to different isolation and titration requirements, it might be beneficial for high-throughput pipelines to allocate different bacterial species to different work teams in steps 2 and 3. High manpower is particularly important for steps 1–3. Titration with three cycles increased the efficiency of our pipeline, as demonstrated by comparing phase II and III of our study (see also Table 1). Further recommendations on every single step of the pipeline are detailed in Section 3.5 of this manuscript.

Interestingly, all phages from our *K. pneumoniae* phage collection showed no matching with the isolated strains of this study, despite >350 phages available in this collection. This can be explained by the high variability of capsule types occurring in *Klebsiella* spp. (at least 78 capsule types) [26]. Additionally, different receptors on the cell surface, such as lipopolysaccharides and outer-membrane-proteins, used for phage entry, must be considered [27].

Genetic safety testing revealed that none of the determined phage genomes carried undesired DNA sequences, such as genes for lysogeny, increase of bacterial pathogenicity, or antimicrobial resistances. Should these genetic modules occur in candidate phages, genetic modifications, such as removal or inactivation of undesired DNA sequences, may be considered, although this approach is currently only rarely in use for personalized phage therapy [28]. Regarding time requirements, Luong et al. indicated five to seven days for step 3 of our pipeline, which was confirmed in our real-life setting (three to six days during phase III) [12]. The preceding and necessary steps 1 and 2 of our pipeline required an additional 48–72 h and 48 h, respectively. The following, step 4, was indicated with three days by Luong et al., which was matched in our study [12]. The production step in our study (step 5a–c) showed that phage production using a cell-free extract system takes only 10 h, if the respective phage can be produced in vitro. These high-quality, cell-free produced phages could be combined with already amplified natural phages to cocktails if desired. In the future, we can expect more safe and high-titre produced phages, using a cell-free extract system. Interestingly, myoviruses had been excluded beforehand from cell-free expression, due to a larger genome and higher metabolic load on the cell-free expression system, as compared to those of other phage families. However, the expression was successful without changing our cell-free system, which has previously only been achieved by Rustad et al., to the best of our knowledge [20]. In summary, the pipeline presented here for phage delivery directly using freshly isolated phages, currently offers the greatest chance of matching to the bacteria of interest. However, this personalized approach is not suitable for patients with a time window of less than two weeks. This underscores the need for internationally accessible, ready-to-use phage banks, possibly partly in lyophilized format, that provide a much larger GMP-phage supply than is currently available [16,29].

### 3.4. Limitations

We assumed that there is always sufficient personnel capacity. Phases II and III, as well as their corresponding E factor calculation, did not include step 4 and 5b, as their standardized protocol did not allow for optimization, and these steps are expensive. Moreover, from experience, host-specific isolation of phages from sewage is easier in summer, such as in our study, than winter. The E factor equation allows comparison of different phage delivery pipelines for different bacteria; however, it does not provide a standardized comparison between different laboratories because parameters such as required volume are not protected from change but will affect the result. This study focused on phage delivery and not on country-specific safety and quality controls, pharmaceutical formulation, or phage administration to the patient. The guidelines are assembled based on decades of phage therapy experiences from international phage experts, but the authors do not take liability for any conflicts that may arise based on these guidelines.

### 3.5. Non-binding Guidelines

The guidelines for personalized phage delivery and therapy, presented here, reflect expert opinions and a framework for all involved parties to ensure high-quality phage preparations and assistance for non-phage experts. These guidelines are not a complete list of methods, but a summary and overview of important steps. All these guidelines are non-binding, and they must be tailored to the individual patient and country-specific requirements. It should be noted that different competencies can be combined in one centre or even in one person. The term patient includes the patient’s legal representatives for decision-making.

#### 3.5.1. Overview

After a clinical request for phage therapy, isolation and antibiotic susceptibility testing of bacteria is carried out in routine diagnostics by clinical diagnostic services (Step 1, see Figure 1).

Bacteria of interest are transported to the phage laboratory, in order to further characterize the bacterial capsule type for a better understanding of the intended phage treatment (Step 2). Particularly in regard to *wzi* capsule types, this step can shorten the time for screening existing phage collections. However, depending on the bacterial species, further bacterial characterization, such as capsule typing, might be omitted. After characterization, bacterial strains can be collected for further use and screened for their phage host range.

Phage isolation, and subsequent phagograms, determine the host range of the phage (Step 3). Phage training could provide more robust phages in this step [30]. After saving part of the phages in a phage collection for future phage applications, phages are transferred to the sequencing laboratory for characterization and a sequence-based safety assessment (Step 4). Production of the phages in high-titres and volume might be carried out in the phage laboratory itself (5a), whereas in vitro (cell-free extract) phage production or genetic engineering might be outsourced to specialized laboratories or companies (5b). GMP production sites offer the opportunity to proceed to a clinical study. All production facilities need to ensure high safety and quality standards. Phages or phage cocktails are prepared in accord with pharmaceutical, medical, and phage specialists. Cocktail preparation sometimes requires short-term preparation before use, as the stability of phages in the cocktails might be dissatisfying [16,31,32,33]. Placing phages in international phage banks offers the great advantage that phages can be rapidly deployed after successful phage-bacteria matching; however, current phage banks do not provide all therapeutically needed phages. According to our practical assessment, patients with a therapeutic window of <13 days might not be able to profit from the isolation of matching phage(s), followed by a safety assessment; however, this might change in the future, and it highly depends on regulatory frameworks. After re-evaluating the patient’s requirements, eligibility, and consent, the phage preparation is applied, and this is followed by close monitoring of clinical and biological parameters. If monitoring reveals bacterial resistance to the administered phage, phages can either be re-isolated (return to step 1) or a new matching procedure can be attempted, using phages from phage banks. This pipeline can be customized to meet individual patient needs and specific local regulations.

#### 3.5.2. Guideline No. 1: Evaluation of Adequacy for Phage Therapy from Four Different Perspectives: Patient, Bacterial Infection, Logistics, and Ethics

Responsible: physicians, ethics department, diagnostic services, phage experts.

The first guideline covers the evaluation of suitability for personalized phage therapy from four different perspectives:

(1) patient’s request for phage therapy,
(2) bacterial infection,
(3) logistics required for phage therapy, and
(4) ethical adequacy.

(1) Patient’s request for phage therapy: A physician trained in phage biology will discuss the risks, in general, as well as possible side effects in detail, and chances of success of phage therapy in a calm atmosphere [34]. Possible misinformation or misunderstandings are addressed in a friendly manner, and the adjuvant nature of most phage therapies is explained. From initial educational discussion to informed consent discussion, patients should have sufficient time for reflection, and they will make the decision out of their own volition. Persuasion to undergo phage therapy is prohibited.

(2) Bacterial infection: The bacterial infection and clinical presentation, including polymicrobial characteristics, time course of the infection, possible therapeutic agents, impact on the patient’s well-being, and availability of phages for therapy against the bacteria of interest, are analysed in detail [35]. For many bacterial infections in humans, lytic phages are not available, or are not available in sufficient numbers, e.g., against *Helicobacter pylori* or *Neisseria gonorrhoeae*.

(3) Logistics: Logistics combining the different competencies required for phage therapy should be assessed and optimized as indicated in this manuscript. Particularly, the following resources should be available:

- Resources for bacterial isolation, such as diagnostic services;
- Transport with a certified courier within 24 h;
- Close contact with an appropriate phage laboratory;
- Resources for phage production in therapeutic quality;
- Storage facilities for phage preparations, including monitoring procedures for storage conditions;
- Facilities for patients and staff for phage therapy itself over possibly long treatment periods (e.g., patient stays on the hospital ward or he/she visits regularly the physician’s office); and
- Monitoring capacities, including:
  ○ diagnostic services for monitoring of the bacterial infection;
  ○ a phage laboratory for phage monitoring; and
  ○ a clinical laboratory for laboratory parameter monitoring.

The adequacy of the logistics needs to be assessed before phage therapy preparation begins [34,36].

(4) Ethical adequacy: The treating physicians inform the responsible Ethics Department about the intended phage therapy, and the Ethics Department issues ethical approval or waives the need for approval. The treating physicians will prepare an individual consent form, in consultation with the Ethics Department. This individual consent form addresses the patient’s individual circumstances and clearly outlines the phage therapy procedure, potential complications, and alternative treatment options. All co-morbidities, pre-existing conditions, allergies, and medication are considered [34].

If treatment was planned under Article 37 of the Declaration of Helsinki [36,37,38], all criteria listed therein must be considered, including the following:

- The patient shows a high degree of suffering.
- The disease does not improve or does not improve sufficiently under conventional therapy.
- The patient is in a hopeless situation.
- Participation in a clinical trial or compassionate use program is not possible.
- Risk assessment was performed by experienced personnel.
- The patient was individually and comprehensively informed of the potential benefits, risks, and availability of alternative therapeutic approaches by experienced personnel prior to treatment, with at least 24 h to consider.

Depending on the requirements of the respective country or state, the regulatory (federal) authorities must be informed about the planned treatment [36]. In addition to the above responsibilities, the involvement of the Clinical Legal Department or legal advice, in general, should be considered.

#### 3.5.3. Guideline No. 2: Properties of the Phage Laboratory and Laboratory Documentation Requirements

Responsible: phage laboratory

Experience:

Laboratory staff for steps 2 (bacterial characterization) and 3 (phage isolation and titration) of the pipeline is routinely working and highly trained with standard phage workflows, such as:

- Titration, plaque, or spot assays for phage-bacteria matches, plaque morphology, change of plaque morphology over time, efficiency of plating, and lysis/growth curves for phage-bacteria matches [18,39,40,41,42,43];
- Effect of antibiotics, polymicrobial infection assays, and phage cocktail performance assessments [15,39,44,45,46];
- Phage training for robust phages [30]; and
- Neutralization assays for assessing anti-phage immune responses [16].

Transmission electron microscopy can provide additional information; however, it is not mandatory prior to phage therapy [11,34,47]. The phage laboratory personnel might be trained in further methods, such as ultracentrifugation, dialysis, biofilm formation, and flow analysis. Phage laboratories can find detailed recommendations in the literature [11,18,39].

Facilities, equipment, and maintenance:

The laboratories for steps 2 and 3 should be equipped with certified, maintained, and regularly inspected sterile working facilities, equipment, and technical devices [11,39]. Monitoring of critical parameters, such as fridge and freezer temperatures, is recommended [11]. The required biosafety containment level depends on the bacteria of interest [11].

Additionally, transport routes are analysed prior to treatment, such as the safe transport of phages or the already extracted DNA to the sequencing laboratory or phage production site.

Documentation for phage laboratory:

All material, equipment, and technical devices are designated to their purpose in a written form, and all validation, maintenance, and inspections are documented [11]. All performed methods of pipeline steps 2 (bacterial characteristics) and 3 (phage isolation and titration) are standardized and issued as standard operating procedures [11]. The results are properly documented in a separate phage therapy file, i.e., when successful or unsuccessful. Abedon provides a detailed description of the relevant parameters that should be analysed and reported [35].

#### 3.5.4. Guideline No. 3: Genetic Safety Check

Responsible: sequencing laboratory

Genetic safety testing, based on the viral sequence, is performed by an expert laboratory on phage genetics, considering the following aspects [11,12,17,34,47,48,49]:

(A) Determination of genes/gene products associated with undesired effects, such as insertion sequences, antimicrobial/biocide/metal resistances, virulence factors, genes with allergenic potential, and toxins.
(B) Evaluation of the predicted phage lifestyle (lytic vs. lysogenic, presence of integrases).
(C) Taxonomic phage classification.
(D) Optional: Evaluation of the transduction potential of phages, based on whole genome sequencing results.

Predicting the suitability of phages for patients can be challenging, and it requires a high level of expertise.

Sequencing will detect prophages if significant amounts are present [30]. Low amounts of prophages may be negligible, compared to, for example, prophage induction by antibiotics [30].

If a bacterium is used for phage production that does not originate from the patient to be treated, the sequencing of external bacteria may be considered if not already performed [11,30,50]. This must be balanced with the therapeutic time window [34] and already existing information, such as phenotypic resistance testing, capsule determination by *wzi* PCR for *Klebsiella* spp., and the ability of the phage to mediate transduction [16]. Where selection between external bacteria for phage production is possible, external bacteria should contain as few toxic, prophage, or phage-like sequences as feasible [11,30].

In principle, it might also be considered to genetically engineer candidate phages for personalized therapy, for example, to remove detrimental determinants or inactive lysogeny modules [28].

Genetic safety evaluation is properly documented, transmitted, and explained to the phage laboratory personnel and treating physicians. It might be part of the safety sheet issued by the production laboratory [11].

#### 3.5.5. Guideline No. 4: Phage Production Choices

Responsible: phage laboratory, specialized phage production site, physicians

Step 5 of the pipeline (phage production) offers three choices:

Step 5a and 5b: Since phage therapy is mostly used for personalized attempts, due to the high specificity of phages and natural co-evolution with the bacterial host, phages are produced and controlled for quality and safety in a phage laboratory (step 5a) or transferred to specialized production facilities (step 5b).

Regulatory requirements for steps 5a and 5b:

The laboratory must meet its country-specific requirements. Regulations might include:

- Production licenses;
- Verified sterile work facilities: from clean benches or space only for phage therapy preparations to clean rooms;
- Ventilation with air quality assessment;
- Documented experience with phage therapy preparations; and
- Compliance to pharmaceutical monographs if available [11,24].

Since most countries do not offer phage specific regulations [19,24,37,51,52], the following overview might help non-phage experts, such as doctors, to assess the quality of a phage production laboratory:

- The production laboratory provides phage expertise in
- Host strain suitability;
- Efficacy testing;
- Bacterial residue assessment and removal including endotoxins;
- Sterility testing;
- Assessment of purity; and
- Conditions for phage storage (cooling, freezing, lyophilization, spray drying, master and working seed lots, and shelf-life monitoring) [11,24,39,53].

Phage identity is assessed in step 4 of the pipeline, and pH determination and adjustment are described in Guideline No. 7. A summary of the important steps for quality and safety control is provided in Guideline No. 5 and detailed publications on this subject [11,24].

Defined working spaces, defined entrance allowances, suitable clothing, written standard operating procedures, risk assessment, and dual control principles for critical process steps are warranted by the production laboratory [11]. Process validation must be successfully finished prior to phage production for patients.

All personnel involved in production, which may include cleaning personnel, are subject to intensive and regular continuing education and practical training about hygiene regulations and process flows. Educational courses and practical training are documented.

Permanently attached labelling of the produced preparations includes:

- Name of the patient and mode of application;
- Name of the prepared preparation, concentration, and volume;
- Storage information;
- Expiry date, if assessed by previous in vitro studies; and
- Name of a contact person involved in the production process [11,24].

The primary packaging material must be evaluated for sterility. The suitability of the primary packaging material for maintaining high-titre phage preparations, for example, with regard to phage adhesion, should be evaluated whenever possible, with regard to the therapeutic window [16,34].

Step 5b: The specialized production laboratory or facility can be an in vitro production facility, based on a cell-free extract system, with the possibility of phage solutions presenting only low levels of endotoxins. Specialized production laboratories or facilities also open possibilities of genetic engineering, such as knock-out of lysogeny genes, if no lytic phage is available for phage therapy.

Step 5c: The GMP standards to be met vary from country to country, and they typically require more than one year and a large financial investment to produce a phage cocktail [52]. This process provides a standardized phage product of the highest quality, and it is, therefore, desirable [16]. In personalized phage therapy, which is discussed in this manuscript, challenges posed by initial and continuous individual matching of phages to bacteria, as well as evolving resistances during treatment, need to be addressed in the same manner for GMP phages as for other phage preparations via enhanced monitoring and re-matching of new phages, if necessary.

#### 3.5.6. Guideline No. 5: Quality and Safety Standards

Responsible: phage laboratory, specialized phage production site, physicians

Quality and safety standards vary widely, depending on country-specific requirements (if available) and the patient’s emergency situation. In particular, the following aspects are considered during purification.

Endotoxin levels: Phage solutions that have been isolated and/or amplified on Gram-negative bacteria might contain endotoxins that are toxic in vitro and in vivo [12,18,54]. According to the European Pharmacopoeia, up to 5 EU/kg/h is permitted for parenteral use (European Pharmacopoeia, 1997) [11,12]. Endotoxins can be removed using several methods, such as LPS affinity chromatography, and numerous kits are available [12,55]. Another smart option is the cell-free system, which requires expertise but delivers high-quality phage, without the need to remove endotoxins [21].

Bacterial or chemical residues: Apart from endotoxins, further bacterial or chemical residues in the phage solution, if present, are removed, and the removal is quantified [11,39]. Bacterial residues cover, for example, exotoxins and lipoteichoic acids [34,56]. A prominent example for chemical residues is cesium chloride (CsCl), which is used for traditional density gradient ultracentrifugation [12,39]. The removal can be achieved with laboratory methods such as dialysis [12,34]. A loss of titre of the phage can be countered with a preceding large-scale titration [57].

Sterility of the phage preparation: Sterility of the phage solution is achieved via standard phage workflows, such as filtering the solution through 0.22 µm filters [11,18,47]. The integrity of the sterile filter must be verified and documented. The sterile filter used must be retraceable via the documentation. Sterility of the phage solution, as well as a visual absence of particle impurities or discolorations, must be confirmed before and during phage therapy [11].

Cell viability assays: Cell viability, upon phage treatment, can help evaluate the toxicity of the phage product [12]; however, the assay is not mandatory, due to a lack of standardized and validated approaches.

Further requirements, from a biological/clinical perspective, that are already covered in other steps of the pipeline, but need to be summarized for the quality statement, are:

Viability, enumeration, and potency of the phage(s) [11]: already confirmed in step 3 of the pipeline and re-confirmed if the phage is produced in steps 5b or 5c.

Identity of the phage(s) [11]: already confirmed in step 4 of the pipeline.

The phage production laboratory or facility issues a safety sheet/declaration, in which all performed steps, from bacterial characterization to phage isolation and amplification, as well as a detailed description of the methods, used reagents, and results, are listed (description of the production process) [11,56].

It is recommended that the treating physicians are trained to understand and evaluate the safety and quality requirements, particularly for individual healing attempts.

It is important to note that the phage laboratory is obliged to immediately inform the treating physicians of any adverse events in the production process, such as resistance or contamination detected.

#### 3.5.7. Guideline No. 6: Re-Evaluation of Adequacy for Phage Therapy from Three Different Per-spectives: Patient, Bacterial Infection, and Ethics

Responsible: physicians and phage experts

Prior to the final formulation and administration of phages, treating physicians reassess the eligibility for phage therapy, regarding patient, bacterial infection, and ethics, see also Guideline No. 1. This reassessment is properly documented.

Particularly, the status of the patient and lack of better therapeutic alternatives, involving microbiological, infectiology, and/or phage expert opinions, is stated. The intended phage therapy is also revaluated, in relation to co-morbidities, pre-existing conditions, allergies, and medication.

If the patient has recovered from the bacterial infection, or if better therapeutic alternatives have emerged, preparations for phage therapy are stopped, regardless of how much effort has already been expended.

#### 3.5.8. Guideline No. 8: Documentation During and After Phage Therapy

Responsible: pharmaceutical department, physicians, phage experts

Four main issues are determined for each individual case, in accord with patient, pharmaceutical experts, physicians, and phage experts, prior to administration, namely:

(1) the route of administration,
(2) phage application as a single phage or cocktail, including the rotation scheme,
(3) the time course of treatment, and
(4) formulation of phage(s).

All four topics, including interdisciplinary discussions, are properly documented.

(1) Possible routes of administration for phage therapy have been published in case reports, and they include, depending on the site of the infection, intravenous, rectal, inhalative, intranasal, oral, intra-surgical, and topical application [34,58,59,60]. The route of administration can be adapted during treatment.

(2) A risk-benefit analysis is made between the administration of single phages and phage cocktails. The latter could extend the effective period of phage treatment; however, the interactions of phages in the cocktail may not be properly understood [6,15,16,32,33,34,46,58]. If a phage cocktail is preferred, different receptor targets/choice of phages with different replicative cycles can be beneficial [45,58,61]. Rotating the phage or phage cocktail to another matching phage or phage cocktail, such as on a bi-weekly basis, may prevent treatment failure. Most importantly, the phage-bacteria combination(s) must match, which is demonstrated using standard phage workflows, such as plaque assays or killing curves [11,16,30,50,52,62].

(3) Based on previous experiences, the phage treatment plan can take up to several months, which must be planned in advance, from a logistical aspect [13,36,37]. The application of phages, e.g., daily, is adjusted to the needs of the patient. Phage treatment plans should not interfere with other treatments.

(4) Little is known about the appropriate phage formulation to be used [54,58,63,64,65,66]. Therefore, pharmacological experts or phage experts, trained in pharmacological properties, decide on the best approach (for fluids usually 0.9% NaCl, which is not suitable for all phages), best pH-value (adapted to the phage and route of administration), and suitable pH-stabilizing buffer (e.g., citrate or trometamol) [57,58]. The homogeneity of the solution is examined [11]. Carriers, such as ointments and hydrogels, and encapsulations, such as alginate polymers, can be useful for various routes of administration [57,58].

#### 3.5.9. Guideline No. 8: Documentation During and After Phage Therapy

Responsible: physicians

Treating physicians must comprehensively document the communication about phage therapy with the patient, interdisciplinary expert discussions, treatment plans, adaptions to the intended treatments, all monitoring steps including (severe) adverse events (SAE) or reactions (SAR), and follow-up measures [67], particularly if treating under the umbrella of Article 37 of the Helsinki Declaration.

Additionally, the documentation can be submitted to the respective health insurance company for reimbursement upon request of the patient.

It is important to note that, given the personalized nature of this treatment, it is important to monitor the patient’s well-being more closely, as compared to standardized treatments. Further reading provides in-depth descriptions of important criteria that should be analysed and reported on phage therapy. [35,59].

#### 3.5.10. Guideline No. 9: Monitoring and Follow-Up

Responsible: phage laboratory, physicians, diagnostic services

Monitoring during phage therapy includes the following tasks:

Monitoring of patient’s parameters, i.e., the patient’s clinical, psychological, and laboratory parameters are monitored closely, such as every other day [34,67]. A medical contact person is provided to the patient.

Monitoring of bacterial infection, i.e., diagnostic services monitor the bacterial infection at regular intervals, e.g., twice a week, if the site of infection permits. If the clearance of the bacterium is assumed, phage therapy is terminated. If switch to another bacterial strain is suspected, phage therapy must be terminated or adjusted accordingly.

Monitoring of phage(s): the match between phage and bacteria is monitored during treatment, e.g., once a week by plaque testing, to prevent treatment with mismatched phage. In vitro studies may not reflect pharmacodynamics and pharmacokinetics in the body, and phages co-evolve with bacteria, allowing bacteria to develop resistance to the administered phages [32,37,52,58,64,68,69]. This underscores the importance of monitoring the match between phages and bacteria and having several suitable phages in stock as a reserve [16,39,62]. Additionally, phage titre of the formulated phage solution is closely monitored by phage experts to avoid treatment with too low concentrations, as titre drops are common with some phages [13,58]. To monitor phage absorption and distribution in the body, as well as phage clearance by the body, pharmacokinetic studies may accompany the treatment [32,64].

Monitoring of formulation parameters, i.e., during the treatment period, the pH-value, homogeneity of the solution, and sterility of the formulated phage preparation should be regularly monitored [11].

It is important to note that, given the personalized nature of this treatment, it is critical to monitor the patient’s well-being more closely, as compared to standardized treatments. Abedon proposed a detailed description of relevant parameters that should be analysed and reported [35].

The frequency of follow-up testing, to analyse recurrent bacterial infections, is adjusted to the patient’s condition. The authors suggest that, at a minimum, adverse events encountered should be included in an internationally accessible registry [24].

## 4. Materials and Methods

### 4.1. Ethics Statement

The study was approved by the Ethics Committee of Klinikum rechts der Isar, Technische Universität München, and it follows the Declaration of Helsinki. Informed consent was waived, due to the retrospective nature of the study.

### 4.2. Inclusion and Exclusion Criteria

Gram-negative bacterial isolates from patients were included if routine diagnostic investigations were complete; however, they were excluded if diagnostic parameters were missing or the patient’s age was less than 18 years.

### 4.3. Bacterial Isolates

Primary microbiological cultures of respiratory samples were isolated at four ICUs and normal wards of the University Hospital rechts der Isar, Technical University of Munich, Germany, and they were cultured on Columbia, Schaedler, and chocolate agar (Becton Dickinson, Sparks, MD, USA). Gram stain identification, species identification (Matrix Assisted Laser Desorption Ionisation-Time of Flight Mass Spectrometry, Bruker Daltonics, Billerica, MA, USA), and automated antimicrobial susceptibility testing, in accordance with EUCAST breakpoints and terminology (VITEK^®^, bioMerieux, Marcy l’Etoile, France), were performed for all pathogens. The capsule type for different *Klebsiella* spp. strains was determined in the phage laboratory using *wzi* PCR, as previously described (Figure S1) [26]. The three study runs (phases I–III) are depicted in Table 2. Bacteria were included, regardless of their antibiotic resistance patterns, as pipeline performance for phage delivery was not to be affected.

### 4.4. Phage Isolation, Genetic Safety Assessment, and Phage Production

Phages were isolated from wastewater via plaque assay on Luria broth agar (Merck, Darmstadt, Germany), as previously described (Supplementary Materials) [70]. Based on previous findings, only phages that could be amplified to 10^7^–10^10^ PFU/mL were transferred via transport of hazardous materials at 4 °C to the sequencing and phage production sites [71,72]. Phage DNA was isolated from lysates via proteinase K/SDS treatment at 56 °C for 2 h, as previously described [73]. Phage genomic DNA libraries were prepared using the Nextera DNA Flex kit (Illumina Inc., San Diego, CA, USA), and a 151 bp paired-end sequencing run was performed on an Illumina NextSeq 500 device (Illumina Inc., San Diego, CA, USA). Raw reads and assembled genome data were used for the safety evaluation of phage genomes (Supplementary Materials). Phages were assembled using a cell-free extract system to overcome endotoxin-related safety concerns, according to the procedure previously described, with the adjustments detailed in the Supplementary Materials [21]. TEM images were prepared as described in the Supplementary Materials.

### 4.5. Efficiency Calculation

Efficiency of the pipeline was calculated, based on: patient data retrieval; effectivity of phage isolation, amplification, and characterization; cumulative time of individual steps of the pipeline; final phage titre; and final phage volume at the end of the pipeline (Equation (1) and Supplementary Materials). The R shiny web application was designed to facilitate the calculation, and it was modelled using R Studio version 4.0.2 (R Foundation for Statistical Computing, Vienna, Austria).

## 5. Conclusions

This study presents phage-host matching, under realistic conditions, embedded in a structured interdisciplinary organizational pipeline for personalized phage delivery. We highlighted the potential improvement points for a high efficiency of phage delivery for phage laboratories, and we offered written guidance for non-phage experts for every single step. If we not only address the phages themselves but also provide a stable pipeline for high-quality phage delivery, we will have a strategy for sustainably combating bacterial infections that are no longer responding, or not sufficiently responding, to antibiotics.

## Figures and Tables

**Table 1:** Efficiency calculation. Time, in hours, indicates hands-on time, transfer time between different institutes, and inevitable waiting periods. Transfer time to the next institute is counted to the respective previous step. Step 1–5a: Steps of the pipeline, see Figure 1. E factor minimum/maximum: efficiency factor, calculated using Equation (1), considering the minimal/maximum cumulative time. E factor mean: mean efficiency factor, calculated using minimum and maximum E factor. Phases I and II focused only on steps requiring optimization, and the results of the standardized steps 4 and 5b of phase I were considered for E factor calculation. Parameters with an asterisk * are integrated into Equation (1).

	Number of Patients	Number of Bacteria	Successful Patient Data Retrieval*	Step 1 (h)	Step 2 (h)	Step 3 (h)	Percentage of Bacteria with High-Titre Phages*	Number of High-Titre Phages/Bacteria*	Step 4 (h)	Percentage of Reliable Genomic Sequences*	Step 5b (h)	Final Titre (PFU/mL)*	Final Volume (mL)*	Cumulative Time (h)*	E Factor Minimum	E Factor Maximum	E Factor Mean
Phase I	25	42	95.5% (42/44)	48–72	48	48–336	87.0% (20/23)	2.4 (55/23)	72	87.3% (48/55)	10	1 × 10^10^	11.0	226–538	0.39	0.92	0.65
Phase II	30	34	100% (34/34)	48–72	48	24–72	58.8% (20/34)	1.1 (37/34)						202–274	0.25	0.34	0.29
Phase III	6	6	100% (6/6)	48–72	48	24–96	83.3% (5/6)	2.0 (12/6)						202–298	0.64	0.86	0.75

**Table 2:** Description of different phases of the study to set up, refine, and validate the phage delivery pipeline.

Time Frames	Aims	Sampling Specifications
Phase I 4 weeks	- to assess the most common bacteria isolated from respiratory infections of critically ill patients - to find and test a suitable pipeline	- four different ICUs of one tertiary care hospital - only tracheal secretion or bronchial lavage - all different bacterial isolates
Phase II 2 weeks	- to refine the low performing components of the pipeline	- all wards of one tertiary care hospital - all different sampling techniques - only *Klebsiella* spp.
Phase III 5 days	- to verify findings about the low performing components of the configured pipeline	- four different ICUs of one tertiary care hospital - only tracheal secretion or bronchial lavage - only *Klebsiella* spp.

## Data Availability

Characteristics of patients, bacteria, and phages, as well as the guidelines for the phage delivery pipeline, are detailed in the manuscript and the Supplementary Materials.

## Supplementary Materials

The following are available online at https://www.mdpi.com/article/10.3390/ph15020186/s1. References [74,75,76,77,78,79,80,81,82,83,84,85,86,87] are cited in the supplementary materials.

## List of Abbreviations

Coronavirus disease 2019 (COVID-19), good manufacturing practice (GMP), intensive care unit (ICU), multidrug-resistant Gram-negative bacteria (MRGN), National Center for Biotechnology Information (NCBI), plaque forming units (PFU), polymerase chain reaction (PCR), transmission electron microscopy (TEM).
