# Supplementary material for: Practical Assessment of an Interdisciplinary Bacteriophage Delivery Pipeline for Personalized Therapy of Gram-Negative Bacterial Infections
Source: Pharmaceuticals (Basel). 2022 Feb 2;15(2):186. doi: 10.3390/ph15020186 (PMC8879309; doi:10.3390/ph15020186)
Supplement: Supplementary file 1 [file pharmaceuticals-15-00186-s001.zip › pharmaceuticals-1525207 - Supplementary Material.pdf]

Practical Assessment of an Interdisciplinary Bacteriophage Delivery Pipeline for Personalized Therapy of Gram-negative Bacterial Infections

Appendix 1

Contents of Appendix 1

Supplementary methods 1
Bacterial isolation and characterization 1
Phage isolation 2
DNA extraction, sequencing, and safety assessment 2
Cell-free phage production 2
TEM images 3
Efficiency calculation 3
Supplementary tables 3
Table S1: 3
Table S2: 4
Table S3: 4
Table S4: 4
Supplementary figure 6
Figure S1: 6
References 6

Supplementary methods

Bacterial isolation and characterization

All bacteria from phase I were isolated from tracheal secretion or bronchial lavages of COVID-19 patients or non-COVID-19 patients in the ICU. SARS-CoV-2 infection had been confirmed or excluded via PCR in routine diagnostics. SARS-CoV-2_N1 and SARS-CoV-2_N3 primers were applied, and probe sets for amplification were used on an ABI 7500 real-time PCR cycler (Thermo Fisher Scientific, Darmstadt, Germany) following the protocol of the Division of Viral Diseases, National Center of Immunization and Respiratory Diseases, Centers for Disease Control and Prevention, Atlanta, USA (accreditation authority No. D-ML-14063-02-00) [74]. To refine the low performing components of the pipeline (steps 2 and 3), a second collection period with the most common isolate of phase I from all different wards and collection sites was conducted. A short third study phase that focused on the most frequent isolate of phase I was performed only on ICU samples and during lockdown, and the workflow was verified in the now configured pipeline subsections. All isolates were safely transported to the phage laboratory through licensed hazardous materials transport.

Capsular types:

The capsule type for different *Klebsiella* spp. strains was determined in the phage laboratory using *wzi*-PCR according to Brisse *et al.* [26] *wzi* is a gene conserved in all capsule types of *K. pneumoniae* that codes for an outer membrane protein involved in capsule attachment to the cell surface [74]. It allows a differentiation of the capsule type analogous to the serotype of *K. pneumoniae.* PCR results were verified via agarose gel electrophoresis (Embi Tec, San Diego, CA, USA), and PCR products of the expected size (580 bp) were extracted using a gel extraction kit (Qiagen, Venlo, Netherlands). Extracted PCR products were sequenced by Eurofins Genomics (Ebersberg,

Germany), and capsule types were determined using blast-suite [75] (blastN) of the National Center for Biotechnology Information [76]. *Wzi* capsule types of *Klebsiella pneumoniae.* are shown in supplementary figure 1 (page 6).

Phage isolation

In short, surface/sewage water was centrifuged at 10,000 x g at 4 °C for 10 min to remove sediments from the supernatant. Subsequently, the supernatant was filtered through a 0.22 µm filter with 25 mm diameter (Sartorius, Göttingen, Germany) to remove all bacteria. The filtrate was incubated at 37 °C for 3 h with different *Klebsiella* spp. isolates in Luria broth (Merck, Darmstadt, Germany) to grow high-titre lysates. The lysates were centrifuged again, and they were filtered as described above. Matching phages were identified, and phage concentrations were assessed via plaque assay.

DNA extraction, sequencing, and safety assessment

Raw reads were subjected to the Aquamis pipeline [77] for trimming (fastp [78]) and SPAdes *de novo* assembling (shovill [79]). Aquamis also performs mash (version 2.1) [80] for reference search [81] as well as quast (version 5.0.2) [80] for assembly quality assessment. Genome annotation was conducted using the annotation tool of PATRIC database [82] (version 3.6.2). Further bioinformatic analysis was conducted using the blast-suite (i.e., blastN, blastP) [75] of the National Center for Biotechnology Information [76]. ABRicate [83] was used for screening of antimicrobial resistance genes using the NCBI amrfinder database [84]. Virulence-associated genes were identified using the database of Virulence Factor Database [85] (last access date: Oct 8, 2021).

Cell-free phage production

DNA preparation for *in vitro* production:

The previous prepared phage stock (10^7-10^{10} PFU/mL) was mixed in a 1:1 ratio with Roti-Phenol/Chloroform/Isoamyl alcohol (pH 7.5-8.0, Carl Roth, Karlsruhe, Germany) in a 5PRIME Phase Lock GelTM tube (Quantabio, Beverly, MA, USA). The tubes were inverted several times, and they were centrifuged at 16,000 g at room temperature for 5 min. Following this, two volumes of pure chloroform (Carl Roth, Karlsruhe, Germany) were added to the upper phase of the tube, inverted, and centrifuged at 16,000 g at room temperature for 5 min. The supernatant was transferred to a separate Eppendorf tube. Subsequently, 20 µL of 3M sodium acetate (Sigma Aldrich, St. Louis, MO, USA) and 1 mL of -80 °C pure ethanol (Carl Roth, Karlsruhe, Germany) were added and the tube was stored at – 80 °C for 1 h. The sample was centrifuged (16,000 x g, 4 °C, 30 min,), and the supernatant was discarded. After adding 1 mL of cold 70% (v/v) ethanol (Carl Roth, Karlsruhe, Germany) at -20 °C, a centrifugation step was performed (16,000 x g, 4 °C, 5 min), and the supernatant was discarded again. To evaporate the remaining ethanol, the sample was stored at RT for approximately 15 min. The DNA pellet was then dissolved in 30 µL of nuclease-free water (Thermo Fisher Scientific, Waltham, MA USA).

In vitro phage production:

Phages were assembled according to the procedure described by Rustad *et al.* with the following adjustments [21]. Phage DNA was mixed with a TX-TL system consisting of 6 mM Mg-glutamate, 100 mM K-glutamate, 3 mM DTT, 1.5 mM each amino acid except leucine, 1.25 mM leucine (from RTS amino acid sampler VWR, Ismaning, Germany), 50 mM HEPES, 1.5 mM ATP and GTP, 0.9 mM CTP and UTP, 0.2 mg/mL tRNA, 0.26 mM CoA, 0.33 mM NAD, 0.75 mM cAMP, 0.068 mM folinic acid, 1 mM spermidine, 30 mM 3-PGA, and 4 % PEG-8000 (Sigma Aldrich, St. Louis, MO, USA), which is referred as reaction buffer, as described in Sun *et al.* [86]. A reaction solution of 13 µL consists of 0.40 µL PEG 8000 (64 % w/v, Sigma Aldrich, St. Louis, MO, USA), 0.67 µL dNTPs (25 mM, New England Biolabs, Ipswich, MA, USA), 0.06 µL ATP (500 mM), 6.25 µL reaction buffer, 0.33 µL GamS (150 µM, Biocat, Heidelberg, Germany), 4.75 µL TX-TL and 0.27 µL nuclease free water, which was chilled on ice for 5 min, and this was followed by the addition of 0.27 µL DNA (5 nM). This 13 µL assembly mix was incubated at 29 °C for 8 h to generate phages.

TEM images

The cell-free produced phages were adsorbed on glow-discharged formvar-supported carbon-coated Cu400 TEM grids (FCF400-CU, Science Services, Munich, Germany) for 30 s, followed by a negative stain using a 2% aqueous uranyl formate solution with 25 mM sodium hydroxide for 45 s. Imaging was carried out using a Philips CM100 transmission electron microscope (Philips N.V., Amsterdam, Netherlands) at 100 kV. The images were acquired by an AMT 4-megapixel CCD camera (AMT, Woburn, MA, USA) and imaging was performed at a magnification of × 21000. The images were processed by the plugin Scale Bar Tools for Microscopes for Java-based software ImageJ [87].

Efficiency calculation

Parameters for the E-factor equation of this study are given in Table 1 of the manuscript. For efficiency calculation of phase II and III, steps 4 and 5 were not rerun as the workflows were already optimized and standardized. We made two assumptions regarding the desired time period from bacterial isolation to phage delivery, and required phage titre and volume, all of which are listed below. For other applications and treatment durations, assumptions for the E-factor equation must be adjusted accordingly, in particular, the required volume might be increased if no dilution is foreseen.

Assumption 1: Desired time period: Max. 5 days for high titre (cell-free produced) phages based on clinical experience with superinfected COVID-19 patients.

Assumption 2: Required final phage titre and volume: 1 x 10^{10} PFU/mL and 11 mL, allowing for ongoing monitoring and treatment for at least two weeks with 1 x 10^{9} PFU/mL, e.g., twice daily 1 mL in a nebulizer for non-ventilated patients or in a drug delivery system of metered dose inhalers for ventilated patients.

Supplementary tables

Table S1:

Patient characteristics of the isolates collected during phase I. All included patients in phase I were hospitalized in ICUs, and they presented bacterial infections of tracheal secretions or bronchial lavage with microbiological pathogen identification.

Parameter	Phase I	Phase II	Phase III
Age	Median 70 (range 30-96)	Median 65 (range 45-84)	Median 77 (range 73-90)
Gender female	48% (12/25)	50% (15/30)	50% (3/6)
Antibiotic therapy	100% (25/25)	100% (30/30)	100% (6/6)
COVID-19	68% (17/25)	0% (0/30)	67% (4/6)
In-hospital mortality	44% (11/25)	10% (3/30)	33% (2/6)
Stay on ICU	100% (25/25)	30% (9/30)	100% (6/6)
Length of ICU	Median 27 (range 1-78)	Median 20 (range 1-46)	Median 13 (range 1-44)
Intubation	96% (24/25)	67% (6/9)	83% (5/6)
Bacterial isolation location	Tracheal secretion (30x) and bronchial lavage (12x)	Urine (20x), swaps from different regions (6x), bronchial lavage (3x), blood culture (3x), tracheal secretion (1x), catheter tip (1x)	Tracheal secretion (4x), bronchial lavage (1x), urine (1x)
Infection with different bacteria in the respiratory tract	48% (12/25, comprising 10/17 COVID-19 patients)	Not assessed due to focus only on *Klebsiella* spp.	Not assessed due to focus only on *Klebsiella* spp.

Table S2:

Isolates transferred to the phage laboratory during phase I. Total number=42. *Klebsiella (K.)* spp. included *K. pneumonia* (n=6), *K. variicola* (n=2), *K. oxytoca* (n=7), and *K. aerogenes* (n=2). *Citrobacter* spp. included *Citrobacter freundii* (n=2) and *Citrobacter koserii* (n=2). *Proteus* spp. included *Proteus mirabilis* (n=3) and *Proteus vulgaris* (n=1). *Serratia* spp. included *Serratia rubidiae* (n= 1) and *Serratia marcescens* (n= 1). R=resistant or intermediate resistant. S=susceptible. *one missing value in the respective resistance patterns.

**Bacterial isolates**	**Number of agar plates**	**Number of resistant strains**					
		Ampicillin/ Sulbactam	Piperacillin/ Tazobactam	Ciprofloxacin	Ceftriaxone	Ceftazidime	Meropenem
Klebsiella spp.	17 (40.5%)	11/17	9/17	0/17	0/17	0/17	0/17
Escherichia coli	8 (19.0%)	7/8	5/8	2/7*	1/8	3/8	1/8
Enterobacter cloacae complex	4 (9.5%)	4/4	3/4	0/3*	3/4	3/4	0/4
Citrobacter freundii spp.	4 (9.5%)	3/4	1/3	0/3*	0/4	0/4	0/4
Proteus spp.	4 (9.5%)	1/4	2/4	1/4	0/4	0/4	0/4
Pseudomonas aeruginosa	2 (4.8%)	1/1*	2/2	2/2	1/1*	2/2	2/2
Serratia spp.	2 (4.8%)	2/2	0/2	0/2	0/2	0/2	0/2
Hafnia alvei	1 (2.4%)	1/1	1/1	0/1	1/1	1/1	0/1

Table S3:

Number of isolated phages, phages with titration failure (step 3), and phages without highly reliable phage sequences (step 4) from all phases of this study.

**Phase**	**Bacterial species used for phage isolation**	**Number of phages**	**Number of phages with titration failure**	**Number of phages without highly reliable phage sequences**
Phase I	6x *Klebsiella pneumoniae*	16	1	4
	6x *Klebsiella oxytoca*	24	3	1
	2x *Klebsiella variicola*	5	2	1
	2x *Klebsiella aerogenes*	2	1	1
	4x *Escherichia coli*	11	1	0
	2x *Proteus mirabilis*	2	2	-
	1x *Citrobacter freundii*	5	0	0
Phase II	34x *Klebsiella pneumoniae*	60	23	
Phase III	6x *Klebsiella pneumoniae*	14	2	

Table S4:

Predicted reference phages of step 4 of phase I. Frequency refers to the frequency of prediction within the phages with highly reliable sequences.

**Reference phage**	**Frequency**	**Host bacterium**
Dickeya phage phiDP10.3 clone pD10.contig.26_1 genomic seq (*The phage genome is incomplete. Only a partial sequence could be detected.)*	1	*Klebsiella variicola*
Enterobacter phage phiEap-3	1	*Klebsiella oxytoca*
Escherichia coli phage vB_EcoS-Sa179lw	1	*Citrobacter freundii*
Escherichia phage 2725-N35	1	*Escherichia coli*

Escherichia phage CF2	1	*Escherichia coli*
Escherichia phage EcNP1	1	*Escherichia coli*
Escherichia phage LM33_P1 genome assembly	5	*4x Citrobacter freundii, 1x Escherichia coli*
Escherichia phage vB_EcoM_JB75	1	*Escherichia coli*
Escherichia phage vB_EcoM_OE5505	2	*Escherichia coli*
Escherichia phage vB_EcoS_CEB_EC3a	1	*Escherichia coli*
Escherichia virus PHB17	1	*Klebsiella pneumoniae*
Klebsiella phage AltoGao	1	*Klebsiella oxytoca*
Klebsiella phage K5	1	*Klebsiella oxytoca*
Klebsiella phage KN1-1	1	*Klebsiella oxytoca*
Klebsiella phage Kp_Pokalde_001	1	*Klebsiella oxytoca*
Klebsiella phage KPN4	1	*Klebsiella variicola*
Klebsiella phage NJS1	2	*Klebsiella pneumoniae*
Klebsiella phage NTUH-K2044-K1-1	7	*6x Klebsiella oxytoca, 1x E. coli*
Klebsiella phage P509	2	*Klebsiella oxytoca*
Klebsiella phage PhiKpNIH-10	1	*Klebsiella pneumoniae*
Klebsiella phage P-KP2	1	*Klebsiella oxytoca*
Klebsiella phage SH-KP152226	2	*Klebsiella oxytoca*
Klebsiella phage vB_KpnM_KB57	3	*Klebsiella pneumoniae*
Klebsiella phage vB_KpnP_IL33	3	*Klebsiella pneumoniae*
Klebsiella phage vB_KpnP_KpV74	1	*Klebsiella oxytoca*
Klebsiella phage vB_KpnP_KpV767	2	*Klebsiella oxytoca*
Klebsiella virus KP32 isolate 194	1	*Klebsiella pneumoniae*
Klebsiella virus KpKT21phi1	1	*Klebsiella pneumoniae*
Klebsiella virus PRA33	1	*Klebsiella oxytoca*
Shigella phage SfPhi01 DNA	1	*Escherichia coli*

Supplementary figure

Figure S1:

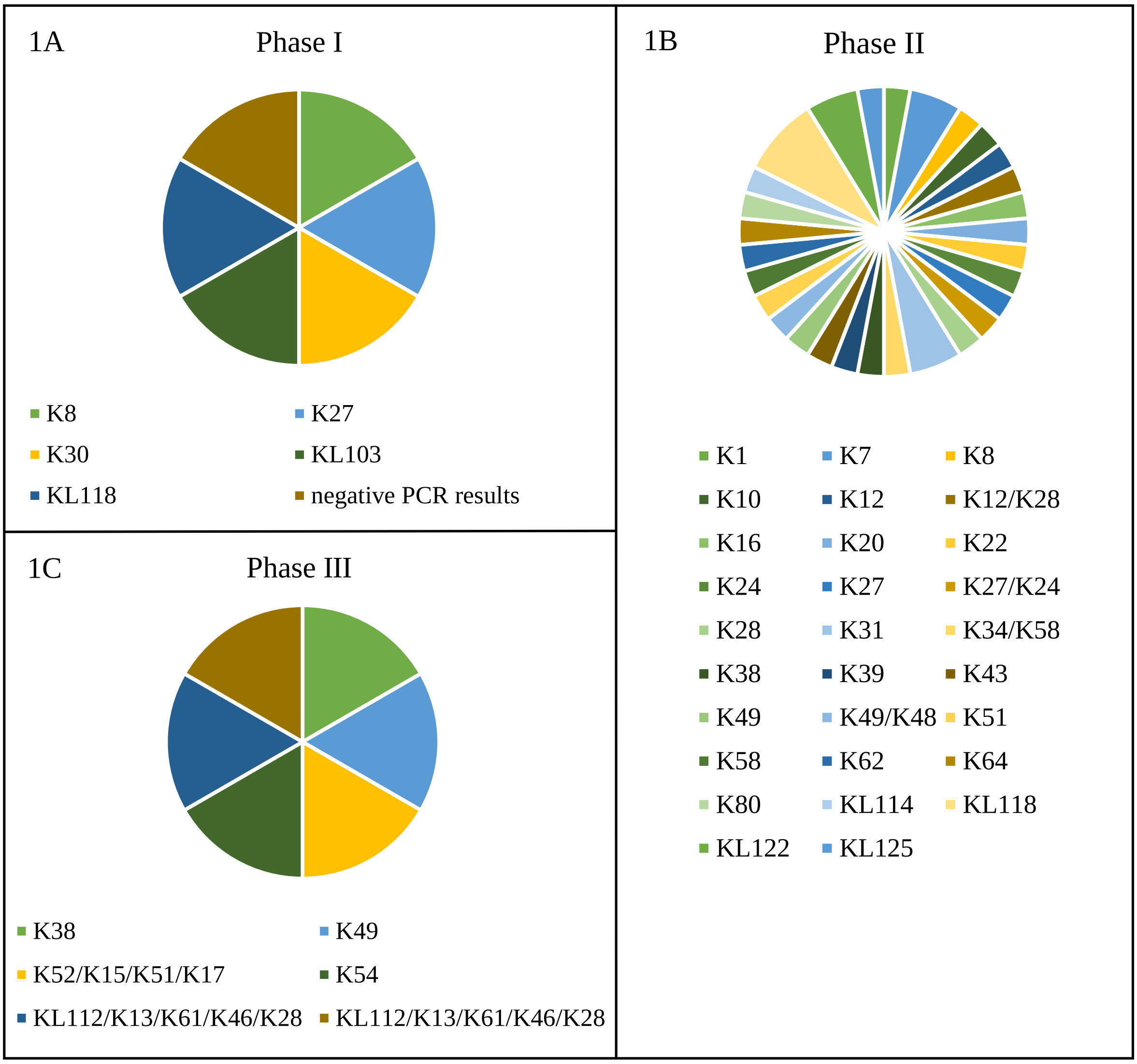

Fig. S1: Homogeneous distribution of *wzi* capsule types of *Klebsiella pneumoniae*. **1A** *wzi* capsule types of *Klebsiella pneumoniae* of phase I of this study, frequency of detection is one for all of six *K. pneumoniae* isolates. **1B** *wzi* capsule types of *Klebsiella pneumoniae* of phase II of this study, frequency of detection is one to three for all of 34 *K. pneumoniae* isolates. **1C** *wzi* capsule types of *Klebsiella pneumoniae* of phase III of this study, frequency of detection is one time for all of six *K. pneumoniae* isolates.

References

References are listed in the manuscript.
